# Remote Antarctic Island reveals unique algal dynamics in snow and ice

**DOI:** 10.1093/ismeco/ycag100

**Published:** 2026-04-22

**Authors:** Emily L M Broadwell, Alexander M C Bowles, Paulina Cifuentes-Uribe, Jasmin L Millar, Daniel Remias, Peter Convey, Christopher J Williamson

**Affiliations:** School of Geographical Sciences, University of Bristol, Bristol, BS8 1SS, United Kingdom; Department of Environmental Science, Aarhus University, Roskilde, DK-4000, Denmark; Department of Biology, University of Oxford, Oxford, OX1 3EL, United Kingdom; School of Geographical Sciences, University of Bristol, Bristol, BS8 1SS, United Kingdom; School of Earth and Environmental Sciences, Cardiff University, Cardiff, CF10 3AT, United Kingdom; Department of Environment and Biodiversity, University of Salzburg, 5020 Salzburg, Austria; British Antarctic Survey, Natural Environment Research Council, Cambridge, CB3 0ET, United Kingdom; Department of Zoology, University of Johannesburg, Auckland Park, 2006, South Africa; Millennium Institute – Biodiversity of Antarctic and Sub-Antarctic Ecosystems, Santiago, Nuñoa 7800003, Chile; School of Biosciences, University of Birmingham, Birmingham, B15 2TT, United Kingdom; School of Geographical Sciences, University of Bristol, Bristol, BS8 1SS, United Kingdom

**Keywords:** glacial microbiology, glacier algae, snow algae, maritime Antarctica, cryptic diversity, novel phenotypes, ecophysiology

## Abstract

Snow and glacier algal blooms are increasingly well-documented in the Northern Hemisphere, but their diversity and ecology in Antarctic regions remain poorly understood. Here, we present the outcomes of high-resolution sampling of snow and glacier algal blooms across Signy Island (maritime Antarctica) during the 2023–24 austral summer (February–April 2024). Using light microscopy and metabarcoding (18S V4 region and ITS2) of environmental DNA, we characterised algal diversity across the frozen habitats available on the island (snowpacks, ice cap, and glaciers), with a relatively long-term (6 weeks) period of ecological monitoring on two contrasting blooms. Our data highlight abundant and diverse snow and glacier algal communities, with snowpacks dominated by the snow algal genera *Sanguina, Chloromonas,* and *Chlainomonas,* extending the range of several species to this region. Ice surfaces supported mixed snow and glacier algal assemblages, including a novel “pointed” *Ancylonema* phenotype alongside described species. Phylogenetic analyses highlighted the presence of amplicon sequence variants representing *Ancylonema* (18S)*, Chloromonas* (18S), and “*Scotiella*” (ITS2) as potentially unique to Signy Island, identifying both cosmopolitan and endemic snow and glacier algal species. Ecological monitoring revealed disparate communities between habitat types, with an atypical névé-type surface recognised as a potentially new ecological niche where *Sanguina* spp. cysts dominated in the presence of glacier algae. Neither community showed evidence of macronutrient limitation. Our work adds to the currently restricted knowledge of these key ecosystem engineers in the Southern Hemisphere and underscores the importance of continued *in situ* studies in these remote environments.

## Introduction

Melting snowpack and glacier ice surfaces are home to diverse microbial communities dominated by snow and glacier algae, which act as the main primary producers and ecosystem engineers [1–3]. During spring and summer melt seasons, extensive algal blooms are formed that can span hundreds of square kilometers [4–7], with cell densities up to 10^3^–10^5^ cells mL^−1^ of meltwater reported across the cryosphere [8–12]. At these concentrations, snow and glacier algae are significant regulators of their own environment, cycling carbon and nutrients into the system [13–18] and exacerbating snow and ice melt through the albedo feedback [4, 15, 19, 20]. Despite this, the current state of knowledge on these unique cold-adapted microorganisms is not proportionate to their ecological significance, in part due to the historic understudy of microbial life in the cryosphere [1, 21]. This has been further compounded by the existential threat that snow and glacier algae face from habitat loss driven by climate change [2], with snowpacks, glaciers, and ice sheets experiencing rapid global decline, with widespread retreat, and in many regions, complete loss expected in just the next 20 years [22]. Maritime Antarctica, for example, is predicted to lose ice cap coverage by 2100 [23–26]. For such regions, which host a high degree of endemism due to geographic isolation and allopatric speciation [27–31], the need to characterise inhabitant snow and glacier algal communities is particularly urgent.

A greater diversity of snow algae, as compared to glacier algae, has been reported across the cryosphere to date, with key chlorophyte snow algal genera including *Sanguina, Chlainomonas, Chloromonas,* and *Raphidonema* [10, 32–39]*.* These more dominant genera can also exist alongside typically less abundant chrysophytes and dinoflagellates within snowpacks [2, 9, 40, 41]. Efforts to constrain snow algal diversity have been hampered by the historic misapplication of names such as *Chlamydomonas nivalis* [41], cryptic diversity between dominant algal taxa [38], and significant heteromorphy of individual taxa between life phases and in relation to environmental drivers [2, 42–44]. Recent advances in molecular approaches have now started to provide a more nuanced view on snow algal biogeography [38, 41, 45–47]. Initial metabarcoding efforts using the 18S V4 hyper-variable region suggested a cosmopolitan distribution of certain snow algal genera [48], but subsequent studies employing faster evolving sequences such as the internal transcribed spacer (ITS2) and ribulose bisphosphate carboxylase large chain (rbcL) indicated the presence of endemism [5, 38]. The first bipolar study of red snow algal bloom communities using ITS2 demonstrated distinct bipolar communities [5], and further work on both ancient and isolated snow algal blooms across the genera *Raphidonema* and *Sanguina,* and the *Chloromadinia* group suggested that modern regional diversity is reflective of the microevolution of ancestral cosmopolitan phylotypes [46, 49]. In Antarctica, research has primarily focused on the Antarctic Peninsula, with studies consistently reporting dominance of *Chloromonas, Chlamydomonas* (possibly now recognised as *Sanguina*)*,* and *Raphidonema* species in coloured snow [32, 35, 50, 51]. Assemblage composition varies between isolated coastal Antarctic snowpacks, reflecting biogeographic structuring in the region [32, 35], with factors such as snowpack structure, nutrient availability, and proximity to wildlife known to influence species’ distributions, suggesting that both local environmental conditions and dispersal processes help shape communities [50–55].

Ice-inhabiting streptophyte glacier algae are currently presumed to be less diverse than snow algae, with *Ancylonema alaskanum, Ancylonema nordenskiöldii,* and *Cylindrocystis brebissonii* being the only fully described species to date. Within the *Ancylonema* genus, *A. alaskanum* has a widespread distribution throughout the European Alps, whereas *A. nordenskiöldii* has been more rarely reported from this region [12, 56, 57]. At higher latitudes, both *A. alaskanum* and *A. nordenskiöldii* have been reported with similar morphological characteristics to those in alpine regions [6, 20, 58–60], with *A. nordenskiöldii* appearing to dominate ice sheet environments [6, 11, 13, 60]. Higher-resolution molecular sequencing is also beginning to better refine glacier algal taxonomy, with recent ITS2 metabarcoding revealing a widespread cryptic diversity within *Ancylonema* when comparing populations across the European Alps and the Arctic [38]. This study also identified the presence of a third, as yet undescribed, *Ancylonema* species within ITS2 metabarcoding datasets from the Swiss Alps and Sweden. In contrast, detailed reports of glacier algal blooms from the Southern Hemisphere remain very sparse, limited to previous work on Tyndall Glacier in Chilean Patagonia [10], and studies from two Antarctic Islands [61, 62]. Metabarcoding datasets were only recently available from the South Shetland Islands [39], showing genetic variation within *Ancylonema* blooms, combined with distinct *Ancylonema* morphologies compared to those widely characterised from the Northern Hemisphere, across the small Robert Island in maritime Antarctica. This study thus confirmed the presence of regional endemism and cryptic diversity within *Ancylonema* in maritime Antarctica, highlighting the requirement for further sampling across the region.

Much of our understanding of snow and glacier algal bloom ecology is derived from well-sampled and accessible sites centered mainly in the Northern Hemisphere [12, 13, 20, 52, 60, 63]. Geographic structuring of snow algal taxa has been highlighted, with factors such as altitudinal zonation [64] and proximity to nutrient sources [65, 66] found to be key drivers in the diversity of alpine snow algal blooms. These microalgal blooms can also undergo a physiological transition over the melt season, shifting from vegetative, flagellated cells to pigment-rich encysted forms. This has been observed for *Chlainomonas* species from selected alpine lakes [42, 67], *Chloromonas* from forest snowpacks [66, 68], and is theorised for *Sanguina* [41, 44]. Alongside shifts in species physiology, species succession has also been documented, typically involving different taxonomic “groups” (often to operating taxonomic units (OTUs)) within the same genera. Such succession has been observed for *Chloromonas* blooms in snowpacks in Japan [68–70] and for *Chlainomonas* blooms on Mt. Rainier, USA [71]. A similar pattern has been reported in the maritime Antarctic, where four coastal blooms dominated by distinct snow algal taxa each progressed through different *Chloromonas* and *Chlamydomonas* groups over the span of a month [54]. For glacier algae, metabarcoding over the season has not yet been conducted, and so it remains unknown as to whether similar taxonomic shifts occur within *Ancylonema* blooms. However, the transition from snow to glacier algal dominance as overlying snowpacks melt and expose bare ice has been reported from Alaska [72] and Iceland [73], suggesting that *Ancylonema* cells are able to colonise habitat as soon as it becomes viable. The co-occurrence of snow and glacier algal species has only been reported within the weathering crust of ice surfaces [39, 60, 61, 72], possibly explained by the ability of *Sanguina* cysts in the region to persist after the recession of the snowline. These studies highlight the complexity of both snow and glacier algal bloom ecology and the need for integrated temporal and taxonomic characterisations, especially for glacier algal blooms in the Southern Hemisphere, where accessibility often limits in-depth temporal studies comparable to those across Arctic and alpine snowpacks and glaciers [37, 67, 72].

Despite these recent advances in studies of snow and glacier algal bloom phenology, progression of these assemblages during the melt season and the influence of taxonomic composition remains poorly understood in the Southern Hemisphere, particularly Antarctica. In this study, we therefore undertook detailed sampling and bloom ecology studies across Signy Island, South Orkney Islands in maritime Antarctica. This location provides the opportunity to examine how algal diversity evolves both across different habitats and throughout the melt season, with snow and glacier algae first reported here in the twentieth century [74, 75]. We performed high-resolution sampling and sequencing of multiple algal assemblages across the island, twinned with detailed growth and photophysiology analyses of selected snow and glacier algal blooms throughout much of the 2024 austral melt season.

## Materials and methods

### Field site and sampling locations

**Figure 1:**
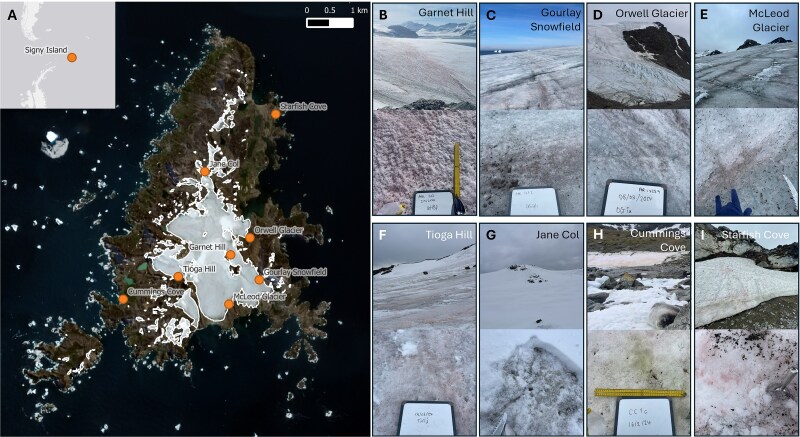
(A) Snowpack and glacier sampling sites across Signy Island, maritime Antarctica; base maps: © ESRI, Satellite images: © Copernicus 2023; (B–I) photographs of the sample sites showing a variety of snow and glacier algal blooms; (B) Garnet Hill, (C) Gourlay Snowfield, (D) Orwell Glacier, (E) McLeod Glacier, (F) Tioga Hill, (G) Jane Col, (H) Cummings Cove, (I) Starfish Cove.

Field sampling to characterise the diversity and ecology of snow and glacier algae on Signy Island in maritime Antarctica took place over February to April 2024 (Supplementary Table S1). Signy Island, part of the South Orkney Islands, is a small (19 km^2^) island with elevations up to 288 m, with approximately half permanently ice-covered by the ice cap and its outlet glaciers. The climate is typical of maritime Antarctica, with less extreme diurnal and seasonal variations than the continent [76]. Mean summer temperatures range from −2°C to 3°C, with strong westerly winds. Eight sites (Fig. 1) were sampled once to cover the diversity of ice cap, valley glacier, and snowpack surfaces present across the island. To assess the variable responses of snow- and glacier-algal-dominated blooms to prevailing environmental conditions, both Garnet Hill and Gourlay Snowfield were sampled weekly for ecophysiology assessment throughout the ablation season from initial snow-covered conditions in early February to track temporal progression in community composition, abundance, and ecophysiology throughout the ablation season (up to 39 and 43 days, respectively).

Prior to the collection of each snow or ice sample, an image with a scale was taken, then the slope, aspect, elevation, and Global Positioning System (GPS) co-ordinates were recorded directly adjacent to the sampling area to avoid contamination using the Phyphox app on an Apple iPhone 12 [77]. Photosynthetically Active Radiation (PAR; μmol photons m^−2^ s^−1^) measurements were taken for the sampling window using a MSC15 spectral light meter with a CSS-45 spectral light detector (Gigahertz-Optik, Germany). For individual snowpack surface samples, a 20 × 20 × 2 cm depth patch of snow was sampled using a presterilised (10% ethanol) metal scoop directly into a sterile Whirl-Pak bag using standard sampling techniques [13]. The same technique was used for individual supraglacial surface ice samples, with the addition of use of a sterilised ice saw to collect the ice.

### Sample processing

Snow and ice samples were melted in the dark over 1–2 days at 4°C and homogenised prior to further processing. An initial 1.5 ml of each homogenised sample was placed into an individual 1.5 mL Eppendorf tube and fixed using Lugol’s solution (1% final concentration) for subsequent cell counts and biovolume estimates at the University of Bristol, UK. A known volume was also filtered through a pre-combusted (450°C for 5 h) 47 mm diameter GF/F filter (0.7 μm retention; Cytiva Whatman, UK), which was wrapped in foil and maintained at −20°C until transport back to the University of Bristol, UK at −80°C on the RRS ‘Sir David Attenborough’. Filters were subsequently stored at −80°C until DNA extraction. Filtrates were collected into pre-acid-washed high-density polyethylene bottles and maintained at −20°C for subsequent inorganic (ammonium, nitrate, nitrite, phosphate) and organic (total organic carbon and nitrogen) aqueous geochemistry quantification at the University of Bristol, UK (Supplementary Data S1, Supplementary Figure S5).

### Molecular analyses

#### DNA extraction

Across all sites sampled, *n* = 3 GF/F filters per site were selected for DNA extraction based on conspicuous algal loading identified by field microscopy. Prior to DNA extraction, filters were lyophilised to break down the cells and aid with DNA recovery. DNA extraction proceeded with a Plant Mini Kit (Qiagen, Hilden) with an initial 15-min shaking step in the presence of glass beads (1 mm diameter, Sigma-Aldrich) and a 30-min incubation at −20°C after the addition of extraction buffer to help lyse the cells [78]. Extraction then continued following the manufacturer’s instructions. DNA recovery was quantified with a Qubit 4 Fluorometer (Thermo Fisher Scientific, UK), and samples with the highest DNA recovery (~2 ng μL^−1^) were then used for sequence amplification.

#### Amplicon library preparation

The 18S V4 hyper-variable region (18S) and internal transcribed spacer region (ITS2) were amplified by polymerase chain reaction (PCR) for sequencing using primers selected from existing literature (Supplementary Table S2). The PCR mixes followed the Illumina “16S Metagenomic Sequencing Library Preparation” guide with minor adaptations from Procházková *et al*. [78] for 18S and ITS2. For 18S, ITS2-s, and ITS2-i, each PCR reaction contained: 10 μL 5× GoTaq Flexi Buffer (Promega, USA), 3 μL DNA template, 1 μL each 10 μM primer, 4.2 μL 25 mM MgCl_2_, 1 μL nucleotide mix (10 mM each), and 0.25 μL 5 μL^−1^ GoTaq Hot Start Polymerase (Promega, USA), made up to 50 μl with nuclease-free water. The cycling conditions used were the same for 18S, ITS2-s, and ITS2-I, with an initial denaturation step at 95°C, followed by 25 cycles of 95°C for 30 s, 55°C for 30 s, and 72°C for 30 s, and a final elongation step at 72°C for 5 min. The library was sent to the Natural History Museum (London, UK) to be sequenced by Illumina MiSeq using the V3 kit (Illumina, USA).

#### Bioinformatic processing

18S (V4 region) and ITS2 gene quality-controlled sequencing reads were processed using the DADA2 pipeline [79] in R [80]. Paired-end reads for each gene were filtered for quality, dereplicated, and denoised as follows: the “filterAndTrim” function was used with parameters set to cut the forward and reverse reads based on the quality scores for each region, with reads with more than two expected errors discarded. The “learnErrors” function was used to estimate the error rates of the remaining reads, which were then denoised using the “dada” function. Both forward and reverse reads were merged using the “mergePairs” function, with a minimum overlap of 12 bp independent for each of the three target regions, producing amplicon sequence variants (ASVs) with a single-nucleotide resolution. ITS2 snow and ice sequence tables were aligned and combined here using the “mergeSequenceTables” function. Chimeras were then removed using the “removeBimeraDenovo” function.

Taxonomic classification of ASVs was conducted on the amplicon using the “assignTaxonomy” function in DADA2 to find the closest match. For 18S sequences this used the Silva SSU 132 database, with the “addSpecies” function then used to compare against GenBank nucleotide sequences for known snow and glacier algae. For ITS2, a custom GenBank nucleotide database of known snow and glacier algae was compiled and used for the identification of ASVs (Supplementary Table S3). The final ASV tables for each marker region were generated and used for subsequent ecological analyses (Supplementary Data S1).

To contextualise the diversity of snow and glacier algae recovered here from Signy Island, comparisons were made with the full breadth of available snow and glacier algal 18S and ITS2 sequence data from Arctic, alpine, and Antarctic environments, including datasets from previous studies [5, 38, 39, 51–54], new sequences generated here from Svalbard and the European Alps (sample site information included in the supplementary), and the full National Center for Biotechnology Information (NCBI) nucleotide database. Basic Local Alignment Search Tool (BLAST) analyses [81] were conducted using established identity thresholds for snow/glacier algae of <99.4% for 18S and <89% for ITS2 to classify sequences (with >60% coverage) as having no matches [38, 82] against the NCBI nucleotide database and published snow and glacier algal sequences [5, 38, 39, 51–54]. For brevity, we present phylogenies using just our newly generated sequences and selected sequences from recently published datasets and the NCBI nt database. Our retrieved ASV sequences were aligned to the wider database using the DECIPHER package [V3; 83]. Tree estimation was subsequently calculated by the neighbor-joining method supported by the phangorn package (V2.12.1) [84], with trees then visualised using iTOL [85]. Non-metric multidimensional scaling (nMDS) plots were determined using Bray–Curtis dissimilarity using ASV sequence abundance from Signy Island, Svalbard, and the European Alps, with significance tested via PERMANOVA.

### Ecophysiological analyses

Cellular abundance (cells mL^−1^) and average biovolume per species (μm^3^ cell^−1^) were assessed for all samples collected in the field. Cellular abundance was measured by counting cells on a modified Fuchs–Rosenthal Haemocytometer (0.2 mm by 1/16 mm^2^; Hawksley, Lancing, UK) using a bright field Olympus BX41 microscope (Japan). Images of each sample were taken at 10× and 40× magnification with a MicroPublisher 6 charge-coupled device (CCD) camera attachment (Teledyne Photometrics, USA) from which cells were counted, and the width and diameter were measured for 15+ cells per species per replicate using ImageJ software [86] according to previously published morphology equations [87]. Final cellular biovolumes were calculated assuming glacier algal cells to be an average cylinder, green snow algal cells as prolate spheroid, spherical snow algal encysted cells to be a sphere, and oblong red snow algal encysted cells to be ellipsoidal [87]. The total algal biovolume per sample (μm^3^ mL^−1^) was calculated by multiplying species’ cellular abundances per sample (cells mL^−1^) by their corresponding mean biovolumes (μm^3^ cell^−1^).

Rapid light response curves (RLCs) [88] were performed using Pulse Amplitude Modulation (PAM) fluorometry to characterise the photophysiological states of the algal communities sampled across Signy Island and the nutrient addition experiments described below. These measurements were conducted on 3 mL subsamples using a Walz Water-PAM fluorometer with an attached red-light emitter/detector cuvette system and stirrer (Walz GmbH, Germany). Each sample was dark-adapted for a minimum of 5 min prior to each RLC measurement, which consisted of nine sequential light steps of 20 s duration ranging in irradiance from 0 to 3000 μmol photons m^−2^ s^−1^ of PAR. The intensity of maximum irradiance was adjusted depending on the algal bloom to achieve photoinhibition.

The maximum quantum efficiency (Fv/Fm) was calculated from minimum (F_0_) and maximum (Fm) fluorescence yields measured in the dark-adapted state during the initial RLC step of 20 s darkness as Fv/Fm = Fm – F_0_/Fm [89]*.* Electron transport through photosystem II (PSII) was calculated across subsequent light steps in relative units (Relative Electron Transport Rate - rETR) assuming an equal division of light between PSI and PSII as rETR = Y(PSII) × PAR × 0.5 [89]. The Eilers and Peeters model was then fitted to the RLCs, enabling the derivation of more photophysiological parameters [90]. These included the relative maximum electron transport rate within PSII (rETRmax), the efficiency of light utilisation (α), and the irradiance at which the electron transport was saturated (Ek).

### Nutrient addition experiments

To examine potential bottom-up controls on the snow and glacier algal communities present on Signy Island, a nutrient spiking experiment was conducted *ex situ*. Samples were collected as above from Gourlay Snowfield (glacier-algal-dominated; 29 February 2024) and Garnet Hill (snow-algal-dominated; 2 March 2024) and melted overnight in the dark. Within 1–2 days after sample collection, two concurrent incubation experiments were established with pooled samples from Gourlay Snowfield and Garnet Hill, respectively, distributed into *n* = 5 replicates across each of five treatments: control (no nutrient addition), +ammonium (+NH_4_^+^, 10 μmol L^–1^), +nitrate (+NO_3_^−^, 10 μmol L^−1^), +phosphate (+PO_4_^3−^, 10 μmol L^−1^), +ALL (+NH_4_^+^ and + NO_3_^−^ at 10 μmol L^−1^ and + PO_4_^3−^ at 2 μmol L^−1^). NH_4_^+^, NO_3_^−^, and PO_4_^3−^ spikes were added as NH_4_^+^-N, NO_3_^−^-N, and PO_4_^3−^-P standards, respectively (Thermo Fisher Scientific, UK). Treatments were chosen to provide ~10-times the reported ambient inorganic nitrogen and phosphorus concentrations in supraglacial ice [91, 92] and to maintain a 10:1 nitrogen:phosphorus ratio within the “+ALL” treatment [59]. Incubations were conducted within 50 mL Corning culture flasks with vented caps (Corning, New York, USA), which were positioned on top of a snowpack throughout the 168-h duration of the experiments, to maintain ambient temperature and irradiance conditions. Sampling of incubations to characterise photophysiological parameters outlined above proceeded 0, 24, 72, 120, and 168 h (only 168 h is shown for brevity).

### Data analysis

R. v.4.4.3 (R Core Team, 2024) was used to analyse and visualise all ecophysiological data. Datasets were tested for normality (Shapiro–Wilk test) and compared statistically using the appropriate test. Parametric data were analysed via a one-way ANOVA, with a subsequent post hoc Tukey Honest Significant Difference applied where appropriate. Nonparametric data were analysed via a Dunn test with a subsequent Kruskal–Wallis test where appropriate.

## Results and discussion

### Novel Southern Hemisphere snow and glacier algal diversity

**Figure 2:**
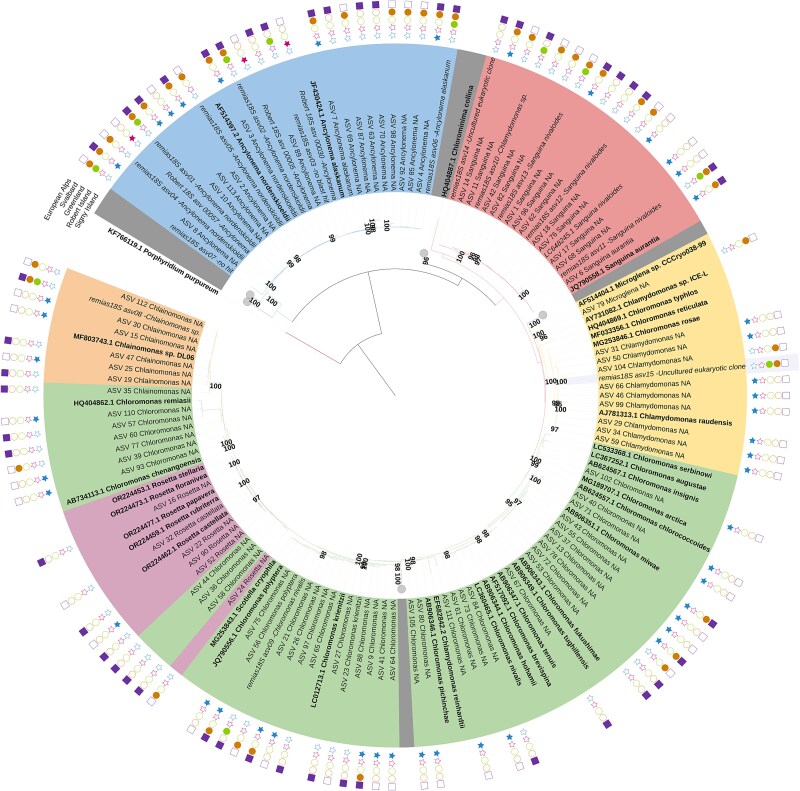
Snow and glacier algal diversity and phylogenetic placement from Signy Island and other studies; maximum likelihood phylogenetic tree for 18S, generated with the GTR + G + I (generalised time-reversible with gamma rate variation) substitution model; statistical support is shown at each node (>95%); reference species are shown in bold with their NCBI GenBank Accession number and species name alongside top 15 sequences in abundance from Remias et al. [38] and select sequences from Thomson et al. [39]; Colours of each taxa indicate genus (blue—*Ancylonema*, red—*Sanguina*, yellow—*Chlamydomonas*, green—*Chloromonas*, pink—*Rosetta*, and orange—*Chlainomonas*); symbols indicate where these ASVs have been reported from (purple square—Alps; this study, orange circle—Svalbard; this study, green circle—Greenland; pink star—Robert Island; blue star—Signy Island; this study).

**Figure 3:**
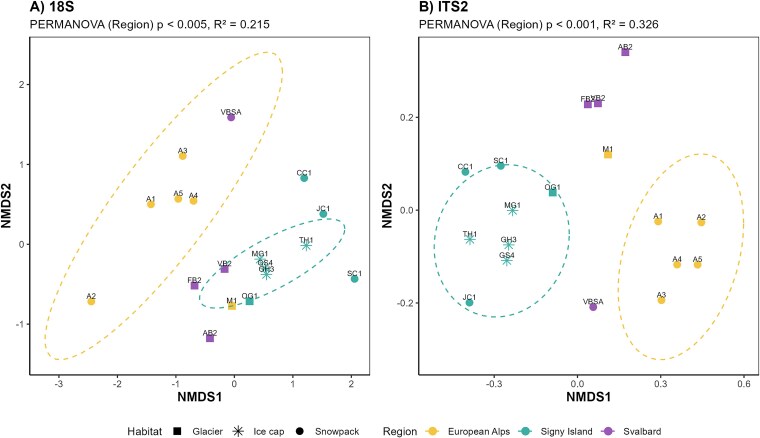
nMDS ordination of (A) 18S and (B) ITS2 community data based on Bray–Curtis dissimilarity (stress values 18S: 0.119; ITS2: 0.069); analysis shows significant differences in the ASV distribution as indicated by a PERMANOVA grouped by region shown by the ellipses (18S: *R*^2^ = 0.215, *P* < .05; ITS2: *R*^2^ = 0.326, *P* < .001).

**Figure 4:**
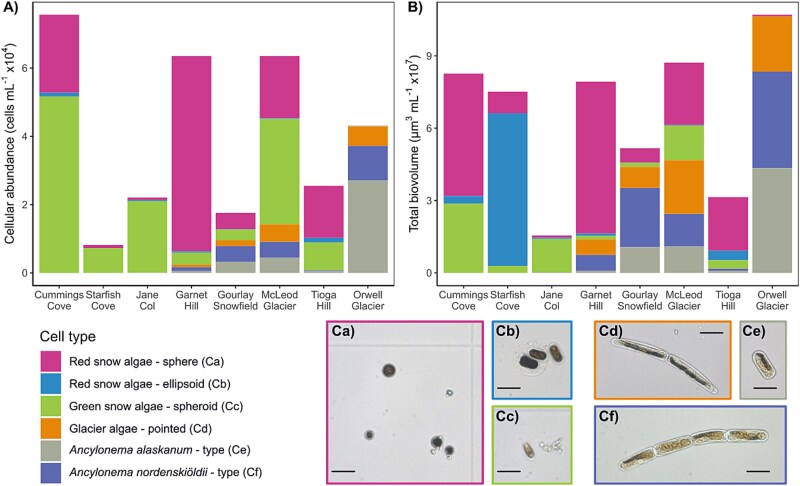
(A) Cellular abundance (cells ml^−1^) and (B) total biovolume (μm^3^ ml^−1^) of the snow and glacier algal species present as determined by the average biovolume per cell per species and cell abundance; bar charts show the mean value (*n* = 10) across various cell types for each sampled location; for the temporal studies, Garnet Hill is represented by Day 18 and Gourlay Snowfield by Day 19 (the same sites as sampled for DNA); (C) fixed microscope images of corresponding cell morphologies to Fig. 2; black bar = 5 μm; (Ca) red snow algae—sphere, (Cb) red snow algae—ellipsoid, (Cc) green snow algae—prolate spheroid, (Cd) glacier algae—pointed, (Ce) *Ancylonema alaskanum*—type, (Cf) *Ancylonema nordenskiöldii*—type.

Abundant and taxonomically diverse snow and glacier algal communities dominated across the breadth of environments studied on Signy Island, Antarctica, during the 2024 austral summer (Figs 1–4). Community composition varied both as a function of habitat type (e.g. snowpacks versus glacier surfaces) as well as between similar habitats (e.g. ice surfaces across the ice cap). Snowpacks overlaying soil and ice surfaces (Cummings Cove, Starfish Cove, Jane Col, Tioga Hill) were colonised exclusively by snow algal assemblages, whilst the ice cap and its outlet glaciers (Garnet Hill, Gourlay Snowfield, McLeod Glacier) showed the presence of both snow and glacier algal taxa. Orwell Glacier, an outlet glacier with its ice surface now detached from the main ice cap (Fig. 1), hosted exclusively glacier algal taxa.

To place the Signy Island communities in a global context, BLAST comparisons were made to available 18S and ITS2 sequence data from across polar and alpine environments. Among the algal sequences recovered across the island, one 18S ASV representing *Ancylonema* and one *Chloromonas*, along with six ITS2 ASVs representing “*Scotiella*” (now recognised as a grouping within *Chloromonas*) had no matches to available sequences (>99.4% for 18S and >89% for ITS2) and so have only been identified on Signy Island to date. Best matches and percentage coverage (>95% unless stated otherwise) for Signy Island snow and glacier algal ASVs (*n* = 52 18S, 122 ITS2) are reported in Supplementary Data; for brevity, we discuss below only the notable aspects of our dataset.

All four *Chloromonas* ITS2 ASVs identified on Signy Island matched with a *Chloromonas* species previously reported from King George Island and Spitsbergen (strains 261-06 and 192-04; CCCryo). However, phylogenetic analysis (Supplementary Fig. S1) revealed overlapping assignments between *Chloromonas* and “*Scotiella*,” reflecting their close relationship [93]. While no 18S ASVs were assigned here to “*Scotiella*,” 23 ITS2 ASVs were retrieved (six unique to Signy).

Of the five 18S ASVs recovered for *Sanguina,* two that were sampled from red snow algal blooms on the ice surfaces at Garnet Hill and McLeod Glacier matched only to sequences from the western Antarctic Peninsula [54]. Notably, *Sanguina aurantia* was identified at Cummings Cove, extending its known distribution beyond the Arctic [38, 41]. For the 13 ITS2 *Sanguina* ASVs found on Signy, four had *Sanguina nivaloides* as their top match and seven had sequences previously reported from Antarctica as *Chlamydomonas* [5]. Eight of these also showed close similarity (~92%) to the novel species DR74a recently recovered from the European Alps and Arctic [38], although it was not a top match for any of them. There were, however, no matches to the other *Sanguina* species (H14) identified in the same study.

For *Chlainomonas*, two 18S ASVs recovered from Signy matched exactly to sequences from the western Antarctic Peninsula [54] and East Antarctica [5]. Of the 10 *Chlainomonas* ITS2 ASVs recovered on Signy, one matched 100% (79% coverage) with sequences recovered from snowpacks in New Zealand [94] and two matched 100% (94% coverage) to sequences recovered from Robert Island [39]. The presence of *Chlainomonas* ASVs at Starfish Cove expands the well-documented alpine distribution of this genus [9, 37, 42, 67] into polar regions, where its biogeography remains almost unstudied [5, 38]. A single *Rosetta* 18S ASV was also recovered in this study from Cummings Cove, matching 100% to unidentified sequences reported from the western Antarctic Peninsula [54] but also matching >99.4% to sequences from the Northern Hemisphere [38]*,* confirming the presence of this recently defined genus within maritime Antarctica [47].

**Figure 5:**
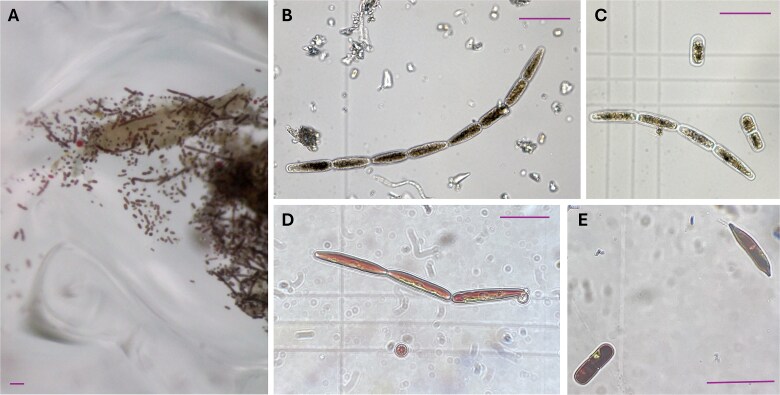
Live and Lugol’s fixed microscope images of the novel *Ancylonema* phenotype; bars = 50 μm; (A) microscope image of surface ice at Orwell Glacier, a mixed *Ancylonema* bloom, (B) long “pointed” filament collected from McLeod Glacier, (C) another filament with evidence of chytrid infection alongside two *Ancylonema alaskanum* type cells from Gourlay Snowfield, (D) live microscope image of a “pointed” cell and conventional “rounded” *Ancylonema* cell collected from Gourlay Snowfield, (E) fixed image of an *Ancylonema* filament with “pointed” apexes next to a *Sanguina* sp. type cyst collected from Garnet Hill.

*Ancylonema* glacier algae were well-represented on Signy Island, with seven 18S and 39 ITS2 ASVs recovered, including one unique 18S ASV sampled from Gourlay Snowfield (Fig. 2 and Supplementary Fig. S1). Notably, ITS2 sequences matching the recently noted “third” *Ancylonema* species [38] were recovered here from Orwell Glacier, extending its known distribution to maritime Antarctica, and the Southern Hemisphere. Metabarcoding of ice samples from Orwell Glacier that were also examined by light microscopy revealed the presence of seven 18S and 39 ITS2 *Ancylonema* ASVs. Though relatively few records exist for glacier algae from this region of the cryosphere, our findings are consistent with previous light microscopy observations from Patagonia [10] and the Antarctic Peninsula [39], which both reported similar pointed glacier algal phenotypes based on microscopy; the latter study recovering three 18S and 20 ITS2 *Ancylonema* ASVs [39] (Fig. 5 and Supplementary Data S1). These data highlight a likely greater *Ancylonema* diversity in our study region than currently understood from the Northern Hemisphere [38], with several ASVs so far unique to Antarctica. All ITS2 ASVs recovered across the island also matched sequences from Robert Island [39], with 19 matching at 100% (92% coverage). While endemism in many snow algal genera has been previously reported [5, 40, 46], it has only recently been signaled for *Ancylonema* spp. [38, 39]. Recent work from Robert Island [39] revealed distinct *Ancylonema* diversity that mirrors the trend shown here on Signy, with glacier algal populations comprising both cosmopolitan and unique haplotypes. Together, our studies are strongly indicative of significant cryptic diversity in Antarctic *Ancylonema* populations, potentially reflecting the region’s role as a long-term “cryo-refugia” for psychrophilic microalgae that once exhibited broader distributions [39].

Bray–Curtis nMDS ordination of 18S and ITS2 sequence datasets of communities sampled for this study in Svalbard, the European Alps, and Signy Island revealed first-order clustering by major geographic region for both amplicons, confirming the distinctiveness of Signy Island assemblages (Fig. 3). Of the 52 18S ASVs and 122 ITS2 ASVs retrieved across these regions, 30 18S and 81 ITS2 ASVs were recovered only from Signy Island. Across both amplicons, communities from Signy were most dissimilar to those from the European Alps, with Svalbard communities displaying an intermediate distribution. This is largely in agreement with previous analyses, which revealed the overlapping nature of snow and ice assemblages across the European Alps and Arctic using 18S (V4 region) and ITS2 amplicons [38], and clear bipolar separation in co-occurrence networks for unique ITS2 snow algal sequences [5]. Bipolar separation has also been observed for other supraglacial communities [29, 95], with Signy Island representing a lower latitude that other bipolar comparisons have missed. PERMANOVA analysis of regional and habitat clustering was more pronounced in ITS2 datasets during this study (*R*^2^ = 0.326, *P* < .001), whilst 18S data showed clustering among glacier-associated communities across all regions (*R*^2^ = 0.215, *P* < .05). This is likely representative of the improved resolution for streptophyte algae offered by the ITS2 marker, providing a better representation of surface ice microalgal assemblages [38, 73, 82]. Snow algal communities from Signy were consistently distinct from their alpine and Svalbard counterparts for both amplicons here, with ice cap samples clustering tightly together and occupying an intermediate position between their co-located snow and glacier communities. Geographic isolation and allopatric speciation likely contribute to the endemism seen here, and previously reported for both snow and glacier algal communities [39, 49], echoing similar patterns observed in other Signy freshwater invertebrates [28] and Antarctic freshwater microalgae more broadly [30, 31, 96]. The extent to which the diversity and distinctiveness of snow and glacier algae observed on Signy and Robert Islands extends more widely across maritime Antarctica remains unknown.

### Abundant and diverse snow and glacier algal communities dominate Signy Island

The distribution of recovered snow and glacier algal taxa across Signy Island was characterised with coupled light microscopy analysis, providing the abundances of key algal phenotypes (Fig. 4 and Supplementary Figure S2). This revealed divergent algal assemblages both as a function of habitat type and over the duration of the melt season. Across sampled snowpacks, *Sanguina* spp. were most abundant at Cummings Cove (38 ± 9%), *Chlainomonas* spp. at Starfish Cove (39 ± 11%), and vegetative snow algae (likely *Chloromonas/Chlamydomonas* spp.) at Jane Col (96 ± 1%). Snow algal abundance was comparable between the coastal sites, averaging 7.56 ± 1.75 × 10^4^ cells mL^−1^ at Cummings Cove and 4.48 ± 1.26 × 10^4^ cells mL^−1^ at Starfish Cove (Fig. 4). Both sites were isolated coastal snowpacks located on soil at low elevations but hosted distinct algal assemblages at the times of sampling. Jane Col, a snowpack located on ice at 150 m elevation, hosted a smaller assemblage averaging 2.20 × 10^4^ (±6.71× 10^3^) cells mL^−1^ (Fig. 4). Another exclusive snow algal bloom dominated by *Sanguina* spp. was observed at Tioga Hill, a more exposed ice surface on the west coast of the island also at 150 m elevation. Total cellular abundances averaged 2.47 × 10^4^ (±5.96 × 10^3^) cells mL^−1^ here (Fig. 4), with the presence of retreating snow patches surrounding this bare ice surface likely accounting for the dominance of *Sanguina* spp. cysts.

Variation in algal assemblages observed across Signy Island snowpacks may have reflected true spatial heterogeneity and/or temporal dynamism in community composition, given that sampling was performed on different days across sites (see Methods). Species successions have been reported in snow algal communities during blooms both in this region [32, 54]; this study and further afield [65, 67]. However, snow algal communities can also display spatial heterogeneity relative to variability in factors such as habitat structure, nutrient availability, and proximity to source populations [32, 49, 52–54, 64, 69, 97]. It is unlikely for macronutrient availability to be responsible for the distinct communities reported here, as coupled aqueous geochemical analysis did not reveal any significant differences between the snowpacks when sampled (Supplementary Data). Longer-term monitoring is required to clarify whether observed patterns represent consistent ecological differences between sites or transient community shifts driven by environmental and seasonal change (e.g. snowpack depth, melt rate, nutrient availability). We show below how both can apply to habitats sampled on Signy Island.

Across the ice surfaces of the Signy Island ice cap (Garnet Hill, McLeod Glacier, Gourlay Snowfield; Figs 1 and 4), the composition and abundance of snow and glacier algal species varied as a function of habitat type. Garnet Hill hosted a distinct red snow algal assemblage dominated by *Sanguina* spp. (Fig. 4), with cellular abundances of 9.60 × 10^4^ (±1.51 × 10^3^) cells mL^−1^, a 10-fold increase on previous *in situ* polar observations [8, 48, 98]. This site was composed of an atypical névé-like ice surface with an almost slush consistency and high water content (Fig. 1B and Supplementary Data Figure S4), similar to that reported from Robert Island [39]. In addition to lower abundances of other snow algal genera (*Chloromonas, Chlainomonas, Chlamydomonas, Stichococcus/Raphidonema*), this site also harboured three distinct glacier algal phenotypes (Figs 4 and 5). This is the first report of *Ancylonema* glacier algae from the South Orkney Islands, with previous reports from maritime Antarctica limited to the South Shetland Islands [39, 62].

In addition to the commonly reported *A. nordenskiöldii* and *A. alaskanum* [3, 57, 99], Signy Island glacier algal communities notably included a striking “pointed” filamentous phenotype that was clearly distinct from these two currently described species (Figs 4C and 5). Cells of this novel glacier algal phenotype (Fig. 5) showed the same dark secondary pigmentation characteristic of *Ancylonema* spp. [57], and were cylindrically shaped with pointed apexes, averaging 12.0 ± 1.6 μm in width and 39.7 ± 15.3 μm in length. Filaments ranged from 2 to ~20 cells and sometimes contained dead cells devoid of pigmentation, with some exhibiting clear chytrid infection [100]. The maximal abundance of this phenotype reached 5.68 ± 1.2 × 10^3^ cells mL^−1^ across all ice surface samples, representing 25 ± 2% of the total glacier algal community and 6 ± 1% of the overall algal community.

McLeod Glacier, characterised by a bare ice surface, hosted mixed snow and glacier algal assemblages, with overall cellular abundance similar to Garnet Hill (6.35 ± 3.22 × 10^4^ cells mL^−1^). Glacier algae were most prevalent here, averaging 1.82 ± 1.00 × 10^4^ cells mL^−1^ (Fig. 4), reaffirming the variability in algal assemblages across Signy. Gourlay Snowfield, a steeper ice surface located a kilometer east of McLeod Glacier and a few hundred meters down-glacier from Garnet Hill, hosted a markedly higher proportion of *Ancylonema* spp. (53 ± 3%) as part of a smaller overall bloom, with total abundances reaching 1.75 × 10^4^ (±4.45 × 10^3^) cells mL^−1^ (Fig. 4).

In contrast to all other sites, Orwell Glacier hosted a near exclusive bloom of *Ancylonema* spp., with a total cellular abundance of 4.28 ± 1 × 10^4^ cells mL^−1^ (Fig. 4). Species composition was dominated by unicellular *Ancylonema* (likely *A. alaskanum*) in terms of cellular abundance, though total biovolume, which also accounts for differences in cell sizes between species [11, 101], was more evenly split between filamentous and unicellular *Ancylonema* phenotypes (61% versus 39%; Fig. 4). This is comparable to the composition of *Ancylonema* blooms reported in the European Alps [12, 56], the Greenland Ice Sheet “dark zone” [11] and in continental Antarctica [61]. Notably, Orwell Glacier hosted the highest abundance and total biovolume of the novel “pointed” *Ancylonema* phenotype, averaging 5.68 ± 1.2 × 10^3^ cells mL^−1^ and 2.29 × 10^7^ (±4.15 × 10^6^) μm^3^ mL^−1^, respectively (Fig. 4). The distinct *Ancylonema* community at this site may be a consequence of the isolated surface conditions of Orwell Glacier as it calves into Cemetery Bay.

### Longer-term snow and glacier algal bloom ecologies

**Figure 6:**
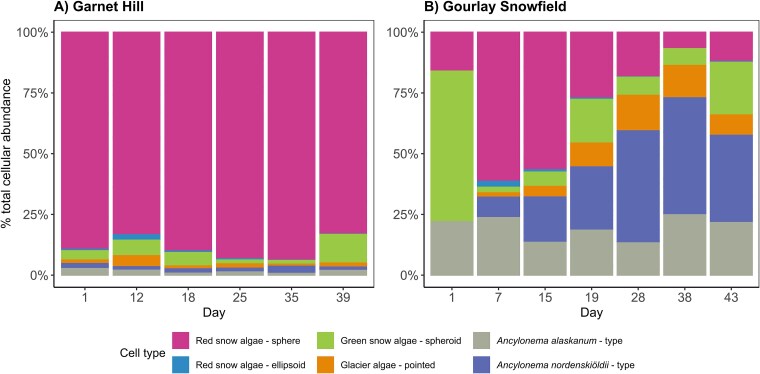
Proportion of total bloom cellular abundance (cells ml^–1^) of each cell type over the 40-day sampling period for (A) Garnet Hill (Day 1 = 13 February 2024) and (B) Gourlay Snowfield (Day 1 = 10 February 2024) as determined by the average biovolume per cell per cell type and abundance; bar charts show the mean proportion (*n* = 10) across each cell type.

Within the atypical névé type surface present at Garnet Hill, community composition remained relatively consistent across the entire 39-day study period (Fig. 6A), with communities dominated by red *Sanguina* spp. cysts (range 75.5%–92.2%), a smaller presence of all three Signy Island glacier algal phenotypes (range 5.5%–11.8% total), and other green flagellate snow algae (range 1.9%–14.5%). While the co-occurrence of snow and glacier algal assemblages has been reported across the Northern Hemisphere [8, 38, 58, 60, 72] and maritime Antarctica [39, 61], it has commonly been attributed to the deposition of cysts from overlying snowpacks following melt events. During repeat sampling on Garnet Hill, no overlying snowpack was present, and only the occasional light rain or snowfall was observed (Broadwell, Personal Observation). Despite this, *Sanguina* spp. cysts maintained a consistently high abundance throughout the 6-week study period (Supplementary Figure S3). This may be explained by the continuous formation of red encysted cells from green flagellates also present in the system, similar to the hypothesised life cycle of *Chlainomonas* [42] and/or a concentration of cells driven by melt of the névé surface, as previously observed in snowpacks [102]. A combination of these processes was recently shown to govern community composition within a snowpack overlying an Alaskan glacier [103]. Notable here is that *Sanguina* spp. cysts remained dominant in this atypical névé surface despite the presence of glacier algae, which may be expected to dominate following the removal of the overlying snowpack [13,72] (see below).

A markedly different and dynamic algal community was present at Gourlay Snowfield as the sampling surface transitioned from frozen snow to melting supraglacial ice over our longer-term observations (Fig. 6B). An initial, relatively low-abundance, green snow algal bloom (Days 1–7) was rapidly overridden by red *Sanguina* spp. cysts, likely delivered to the site from Garnet Hill via a down-slope flushing event preceding Day 12. On Day 19, the community shifted to dominance by *Ancylonema* glacier algae, matching the major transition from snow to bare ice (Supplementary Figures S3 and S4). This confirms that these microbial communities rapidly colonise newly available habitats, utilising the limited windows of favourable growth conditions. In contrast to Garnet Hill, a 3% daily decline in red snow algal cysts and a 9% daily increase in *Ancylonema* abundance were then apparent over the 24-day remainder of the sampling period (Fig. 6B). Thus, a more typical shift in species composition was observed on Gourlay Snowfield [72], tracking evolution of the surface habitat from snow-covered to melting ice.

We interpret our contrasting findings from Garnet Hill to reflect the novelty of its intermediate névé surface, which appears to support a distinct and stable snow and glacier algal assemblage not typically found on snow or ablating glacier ice. Although the névé surface appeared to hold a higher water content (Broadwell, Personal Observation), there were no significant differences in the macronutrient availabilities of the two sites over the course of the sampling period (Supplementary Figure S5). This may point to a previously under-recognised habitat type on ice cap surfaces, reported to date from here and Robert Island [39], that may provide a unique ecological niche, with distinct implications for microbial community composition. This sets maritime Antarctica apart from more widely sampled sites across the Northern Hemisphere, where bare ice surfaces are generally predicted to host glacier algal blooms of increasing density as meltwater availability increases [19, 20, 56, 60]. The dominance of heavily pigmented *Sanguina* blooms on the ice caps of Signy Island and Robert Island [39] of maritime Antarctica suggests that the “Greenlandification” of Antarctica [26] may not have parallel impacts on the snow and glacier algal assemblages in the region.

**Figure 7:**
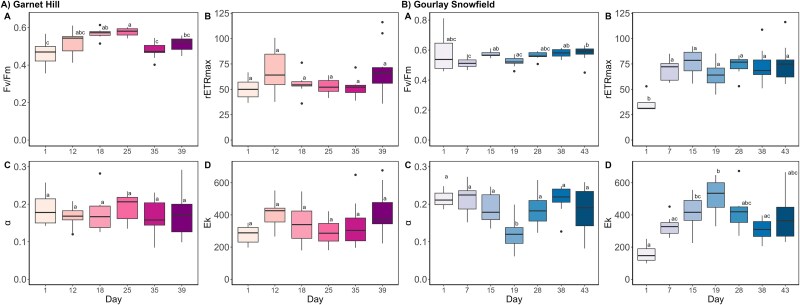
The photophysiology of the (A) Garnet Hill and (B) Gourlay Snowfield assemblages assessed by rapid light curve (RLC) techniques; boxplots show the median, interquartile range, and min to max values, with points showing potential outliers (*n* = 10); lowercase letters denote homogenous subsets; (Aa) maximum quantum yields in the dark-adapted state (Fv/Fm) (KW; *χ*^2^ = 59.69, *P* < .01); (Ab) maximum electron transport rate (rETRmax) (KW; *χ*^2^ = 34.07, *P* < .01); (Ac) efficiency of light use (α) (KW; *χ*^2^ = 29.91, *P* < .01); (Ad) light saturation coefficient (Ek) (KW; *χ*^2^ = 26.15, *P* < .01); (Ba) maximum quantum yields in the dark-adapted state (Fv/Fm) (KW; *χ*^2^ = 24.61, *P* < .01); (Bb) maximum electron transport rate (rETRmax) (F6, 55 = 5.39, *P* < .01); (Bc) efficiency of light use (alpha) (F6, 55 = 5.26, *P* < .01); (Bd) Light saturation coefficient (Ek) (KW; *χ*^2^ = 23.92, *P* < .01).

Parallel tracking of community photophysiology at both longer-term study sites (see Methods) revealed assemblage-specific patterns over the sampling period (Fig. 7). Red snow algal cyst-dominated communities at Garnet Hill exhibited a relatively consistent photophysiology throughout the 39-day observation period, maintaining consistent rates of electron transport (rETRmax), light utilisation efficiency (α), and light saturation co-efficients (Ek). Fv/Fm of the assemblage was consistently elevated, averaging 0.52 ± 0.06. Given the general dominance of this site by encysted *Sanguina* cells that derive their distinctive bright red colouration from extensive accumulation of photoprotective carotenoids [41, 104], it is likely that the photophysiology of these cells remained robust to apparent changes in the light environment. This matches previous reports of stability in red snow-algal-dominated community photophysiology from Mittivakkat glacier, south-east Greenland [60].

In contrast, assemblage structure and acclimation to prevailing environmental conditions shaped community photophysiology at Gourlay Snowfield. In the first instance, the lowest rETRmax and Ek were apparent when green snow algal cells dominated (61 ± 9%) the assemblages (Figs 6B and 7B). Green flagellates lack the accumulation of photoprotective carotenoids characteristic of encysted snow algal cells [42, 105, 106] or photoprotective purpurogallin pigments enriched in glacier algae [20, 99]. Given that their maximal abundance at Gourlay Snowfield (Day 1 of sampling) coincided with the lowest rETRmax and Ek observed, it is likely that the presence of green flagellates in the community translated into increased susceptibility to photoinhibition under the naturally high irradiance conditions prevailing [52, 63, 101, 107]. Subsequent flushing of red encysted snow algal cells into Gourlay Snowfield saw a recovery in community photophysiology, with increasing rETRmax and Ek reflecting the higher light adaptation of these cysts [93,108] (see above).

With dominance of Gourlay Snowfield by *Ancylonema* glacier algae from Day 7 onwards (Fig. 6B), a consistent rETRmax was achieved through dynamic acclimation to changes in the prevailing light environment (Fig. 7B). This manifested as a decrease in α and increase in Ek in response to higher light intensities that peaked on Day 19 of observations (Supplementary Data), during a period of warmer and clearer weather (Fig. 7; Broadwell, Personal Observation). With return to previous conditions after Day 19, reversals in α and Ek were apparent. These data reflect classic photoacclimation to increased irradiance [88, 109] and support assertions that glacier algae show dynamic regulation of their photophysiology [60], despite being enriched in photoprotective pigments similar to encysted *Sanguina* species [20]. This has been attributed to their vegetative life history phase [60], which affords the greatest ability for maximising growth and reproduction during their relatively short summer period [110].

**Figure 8:**
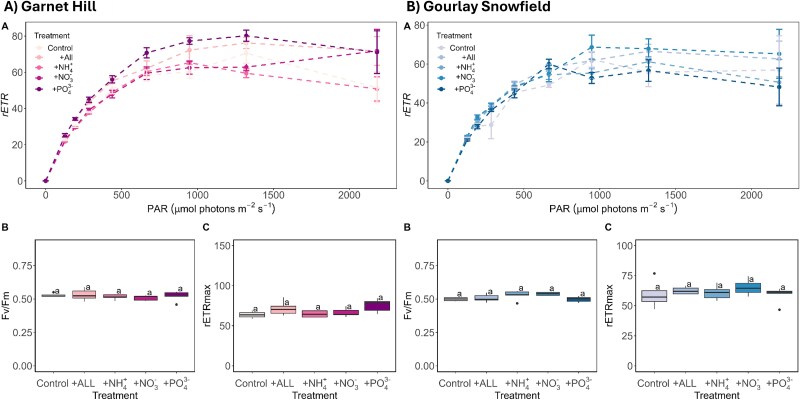
Photophysiology of (A) Garnet Hill and (B) Gourlay Snowfield after 168 h of incubation under the range of nutrient treatments assessed by RLC techniques; boxplots show median, interquartile range, and minimum to maximum values, with points showing potential outliers (*n* = 5); lowercase letters indicate homogenous subsets determined through a one-way ANOVA; (A) RLC traces of relative electron transport rates (mean ± SE, *n* = 5), (B) maximum quantum yield in the dark-adapted state (Fv/Fm), (C) maximum electron transport rate (rETRmax).

Consistent with the adaptation of snow and glacier algae to their low-nutrient environments [11, 16, 17, 101, 111], neither the glacier-algal-dominated assemblage at Gourlay Snowfield nor the snow-algal-dominated assemblage at Garnet Hill demonstrated any notable signs of macronutrient (nitrate, ammonium, phosphate) limitation in their photophysiology during 5-day nutrient-spiking incubations conducted on Signy Island (Fig. 8). Recently termed the oligotrophic-bloom-paradox [12], this is strongly aligned with observations across the Northern Hemisphere, where glacier algal blooms in the Arctic [59] and European Alps [12] showed no indication of macronutrient limitation at the point of sampling. Other studies for coastal snow algal blooms in the region also reported that macronutrient availability did not play a major role in the structuring of the community [53, 54]. This indicates that the macronutrients required to sustain diverse and abundant snow and glacier algal blooms are available across the cryosphere from the Arctic to Antarctic environments. As warming continues to unlock more of the global cryosphere [23, 25, 112], our data add to the growing consensus that an increase in the extent of snow and glacier algal blooms can be expected into the future [4, 15, 17, 59, 65, 113]. Given their potential to establish positive feedback with snow and ice albedo and melt [7, 19, 56, 98], this has serious implications for the future integrity of snowpacks and supraglacial ecosystems. The impacts of the rapidly changing cryosphere on the ecology of snow and glacier algal species themselves remain to be seen.

## Conclusion

Expansive spatiotemporal characterisation of snow and ice habitats across Signy Island, Antarctica, revealed abundant and diverse snow and glacier algal communities with distinct habitat-specificity. Snow-algal-dominated coastal snowpacks, snow overlying glaciers during early melt, and an atypical névé-type ice cap surface that appeared to provide a unique ecological niche. Glacier algae were equally abundant on Signy, dominating supraglacial ice surfaces. Our data extend the known distributions of several snow algal species into the Southern Hemisphere and confirm novel glacier algal diversity within this region, with multiple snow and glacier algal sequences failing to match with any available 18S or ITS2 reports from across Arctic, Antarctic, or alpine environments. Long-term bloom monitoring at Garnet Hill and Gourlay Snowfield identified strong habitat-specific patterns in community composition and associated ecophysiology. In addition to providing substantive new knowledge on snow and glacier algal communities and their ecologies from this region of the cryosphere, our study highlights the need for more thorough sampling of the Southern Hemisphere if the true diversity of snow and glacier algae is to be constrained into the future.

## Supplementary Material

Supplementary_Material_ycag100

## Data Availability

The raw Illumina sequence datasets have been submitted to the GenBank as a SRA project (Accession number PRJNA1321797). Nucleotide sequences have been uploaded to GenBank under the accession numbers PX216263—PX216377 (18S) and PX216003—PX216262 (ITS2) and are available in fasta format alongside the BLAST comparison tables at 10.5281/zenodo.17086497 (Zenodo). All field datasets (abundance, biovolume, rapid light curves, and aqueous geochemistry) are also available at 10.5281/zenodo.17086497 (Zenodo).
