# Supplementary material for: Remote Antarctic Island reveals unique algal dynamics in snow and ice
Source: ISME Commun. 2026 Apr 22;6(1):ycag100. doi: 10.1093/ismeco/ycag100 (PMC13196586; doi:10.1093/ismeco/ycag100)
Supplement: Supplementary_Material_ycag100 [file supplementary_material_ycag100.zip › signy_supplementary_data.pdf]

# Remote Antarctic Island Reveals Unique Algal Dynamics in Snow and Ice

Authors: *Emily LM Broadwell[1,2], Alexander MC Bowles[3], Paulina Cifuentes-Uribe[1], Jasmin L Millar[4], Daniel Remias[5], Peter Convey[6-8], and Christopher J Williamson[1]

1. School of Geographical Sciences, University of Bristol, UK
2. Department of Environmental Science, Aarhus University, Denmark
3. Department of Biology, University of Oxford, UK
4. School of Earth and Environmental Sciences, Cardiff University, UK
5. Department of Environment and Biodiversity, University of Salzburg, Austria
6. British Antarctic Survey, Natural Environment Research Council, UK
7. Department of Zoology, University of Johannesburg, South Africa
8. Millennium Institute – Biodiversity of Antarctic and Sub-Antarctic Ecosystems, Santiago, Chile
9. School of Biosciences, University of Birmingham, UK

**Supplementary Table 1:** Sample location data (N = 10, TS = Time Series).

| Environment | ID | Location | Date | Time | Latitude | Longitude | Altitude (m) | Slope (°) | Aspect (°) | Description |
|---|---|---|---|---|---|---|---|---|---|---|
| Ice cap | GS1 | Gourlay snowfield | 10/02/24 | 12:16 | -60.721088 | -45.6081 | 87.7 | 8.23 | 102 | Outlet glacier from the ice cap. TS. |
| Ice cap | GH1 | Garnet hill | 13/02/24 | 15:46 | -60.716186 | -45.6195 | 238.8 | 7.95 | 158 | Ice cap surface. TS. |
| Snowpack | CC1 | Cummings cove | 16/02/24 | 13:04 | -60.724901 | -45.6624 | 5.5 | 11.62 | 217 | Coastal snowpack on rock/soil. |
| Ice cap | GS2 | Gourlay snowfield | 17/02/24 | 15:27 | -60.721588 | -45.6077 | 84.3 | 10.79 | 119 | Outlet glacier from the ice cap. TS. |
| Ice cap | GH2 | Garnet hill | 25/02/24 | 14:06 | -60.716953 | -45.6183 | 178.6 | 8.49 | 139 | Ice cap surface. TS. |
| Ice cap | GS3 | Gourlay snowfield | 25/02/24 | 14:48 | -60.720876 | -45.6089 | 85.5 | 8.2 | 92 | Outlet glacier from the ice cap. TS. |
| Ice cap | MG1 | McLeod glacier | 28/02/24 | 11:54 | -60.725879 | -45.6202 | 59.2 | 5.91 | 156 | Outlet glacier from the ice cap. |
| Ice cap | GS4 | Gourlay snowfield | 29/02/24 | 13:34 | -60.721259 | -45.6087 | 85 | 6.94 | 120 | Outlet glacier from the ice cap. TS. |
| Ice cap | GH3 | Garnet hill | 02/03/24 | 11:36 | -60.715637 | -45.6196 | 210 | 12.78 | 178 | Ice cap surface. TS. |
| Snowpack | SC1 | Starfish cove | 07/03/24 | 10:58 | -60.688588 | -45.6015 | 14.1 | 31.6 | 135 | Coastal snowpack on rock/soil. |
| Glacier | OG1 | Orwell glacier | 08/03/24 | 12:05 | -60.712864 | -45.6118 | 30.2 | 20.08 | 75 | Valley glacier (previously an outlet glacier but calving away). |
| Ice cap | GS5 | Gourlay snowfield | 09/03/24 | 13:02 | -60.72089 | -45.6087 | 84.6 | 7.15 | 88 | Outlet glacier from the ice cap. TS. |
| Ice cap | GH4 | Garnet hill | 09/03/24 | 14:11 | -60.715674 | -45.6196 | 200.7 | 7.76 | 207 | Ice cap surface. TS. |
| Ice cap | TH1 | Tioga hill | 13/03/24 | 11:18 | -60.720462 | -45.6404 | 179.9 | 11.42 | 215 | Outlet glacier from the ice cap. |
| Ice cap | GH5 | Garnet hill | 19/03/24 | 11:18 | -60.715598 | -45.6199 | 215.8 | 8.59 | 165 | Ice cap surface. TS. |

| Environment | ID | Location | Date | Time | Latitude | Longitude | Altitude (m) | Slope (°) | Aspect (°) | Description |
|---|---|---|---|---|---|---|---|---|---|---|
| Ice cap | GS6 | Gourlay snowfield | 19/03/24 | 12:12 | -60.720882 | -45.6089 | 82.4 | 8.87 | 96 | Outlet glacier from the ice cap. TS. |
| Ice cap | JC1 | Jane col | 23/03/24 | 14:38 | -60.699875 | -45.6298 | 146.8 | 11.32 | 52 | Snowpack on rock/soil located on a slope above the ice cap. |
| Ice cap | GH6 | Garnet hill | 24/03/24 | 11:17 | -60.715656 | -45.6194 | 220.3 | 7.45 | 154 | Ice cap surface. TS. |
| Ice cap | GS7 | Gourlay snowfield | 24/03/24 | 12:28 | -60.721149 | -45.6088 | 92 | 5.21 | 87 | Outlet glacier from the ice cap. TS. |

**Supplementary Table 2:** Primer pairs used for the amplification of the 18S and ITS2 snow and ice marker regions.

| **Amplicon** | **Primer** | **Direction** | **Sequence (5' to 3')** | **Source** |
|---|---|---|---|---|
| 18S SSU rRNA V4 region | 528F | F | GCGGTAATTCCAGCTCCAA | Cheung et al. (2010) |
| | 706R | R | AATCCRAGAATTTCACCTCT | |
| ITS2-s | 5.8SbF | F | GATGAAGAACGCAGCG | Remias et al. (2023) |
| | ITS4 | R | TCCTCCGCTTATTGATATGC | Innis et al. (1990) |
| ITS2-i | 5.8SbF2 | F | CGATGAAGAACGCAGCG | Mikhailyuk et al. (2008) |
| | LSULP | R | AATTCGGCGGGTGGTCTTG | Remias et al. (2023) |

**Supplementary Table 3:** NCBI GenBank reference species included in phylogenetic analysis

| | **18S** | **ITS2** |
|---|---|---|
| *Ancylonema alaskanum* | JF430424.1 | OQ234976.1 |
| *Ancylonema nordenskioeldii* | AF514397.2 | OL898470.1 |
| *Chlainomonas kolii* | N/A | N/A |
| *Chlainomonas rubra* | N/A | N/A |
| *Chlainomonas sp. DL06* | MF803743.1 | MF803744 |
| *Chlamydomonas raudensis* | AJ781313.1 | AJ781314.1 |
| *Chlamydomonas reinhardtii* | EF682842.2 | AB511842.1 |
| *Chlamydomonas sp. ICE-L* | AY731082.1 | AY731085 |
| *Chlorominima collina* | HQ404887.1 | MW554521.1 |
| *Chloromonas arctica* | MG189707.1 | MG189706 |
| *Chloromonas augustae* | LC367252.1 | PP826665.1 |
| *Chloromonas brevispina* | AF517092.1 | MG791868.1 |
| *Chloromonas chenangoensis* | AB734113.1 | LC360497.1 |
| *Chloromonas chlorococcoides* | AB624557.1 | AB624573.1 |
| *Chloromonas fukushimae* | AB906343.1 | AB906384.1 |
| *Chloromonas hohamii* | AB906344.1 | AB906394.1 |
| *Chloromonas insignis* | AB624567.1 | HQ404868.1 |
| *Chloromonas krienitzii* | LC012713.1 | LC012757.1 |
| *Chloromonas miwae* | AB906351.1 | LC012763.1 |
| *Chloromonas nivalis* | LC360465.1 | GU117576 |
| *Chloromonas pichinchae* | AB906346.1 | LC012761.1 |
| *Chloromonas polyptera* | JQ790556.1 | OL898471 |
| *Chloromonas remiasii* | HQ404862.1 | LC360494.1 |
| *Chloromonas reticulata* | MF033356.1 | AB022530.1 |
| *Chloromonas rosae* | MG253846.1 | AF517073.1 |
| *Chloromonas serbinowi* | LC533368.1 | AB624579.1 |
| *Chloromonas sp. CCCryo192-04* | HQ404880.1 | N/A |
| *Chloromonas sp. CCCryo261-06* | HQ404889.1 | N/A |
| *Chloromonas tenuis* | AB906347.1 | AB906395.1 |
| *Chloromonas tughillensis* | AB906349.1 | AB906393.1 |
| *Chloromonas typhlos* | HQ404869.1 | N/A |
| *Cylindrocystis brebissonii* | AF115439.1 | MT796587.1 |
| *Microglena sp.* CCCryo038-99 | AF514404.1 | N/A |
| *Porphyridium purpureum* | KF766119.1 | DQ308439.1 |
| *Raphidonema sempervirens* | AF514410.2 | AJ431674.1 |
| *Rosetta castellata* | OR224462.1 | OR247833 |
| *Rosetta floranivea* | OR224473.1 | OR247792 |
| *Rosetta papavera* | OR224477.1 | OR247815 |
| *Rosetta rubriterra* | OR224459.1 | OR247843 |
| *Rosetta stellaria* | OR224453.1 | OR247818 |
| *Sanguina aurantia* | JQ790558.1 | MZ955646.1 |
| *Sanguina nivaloides* | LC648245.1 | MK733635.1 |
| *Scotiella cryophila* | MG253843.1 | MG253844.1 |

**Supplementary Figure 1:** Max likelihood phylogenetic tree for ITS2�Statistical support is shown at each node and was generated from a GTR + G + I (Generalised time-reversible with Gamma rate variation) maximum likelihood tree. Reference species are shown in bold with their NCBI Gen Bank Accession number and species name alongside top 15 ITSsnow and 15 ITSice sequences in abundance from Remias et al. (2023) and select sequences from Thomson et al. (2025).

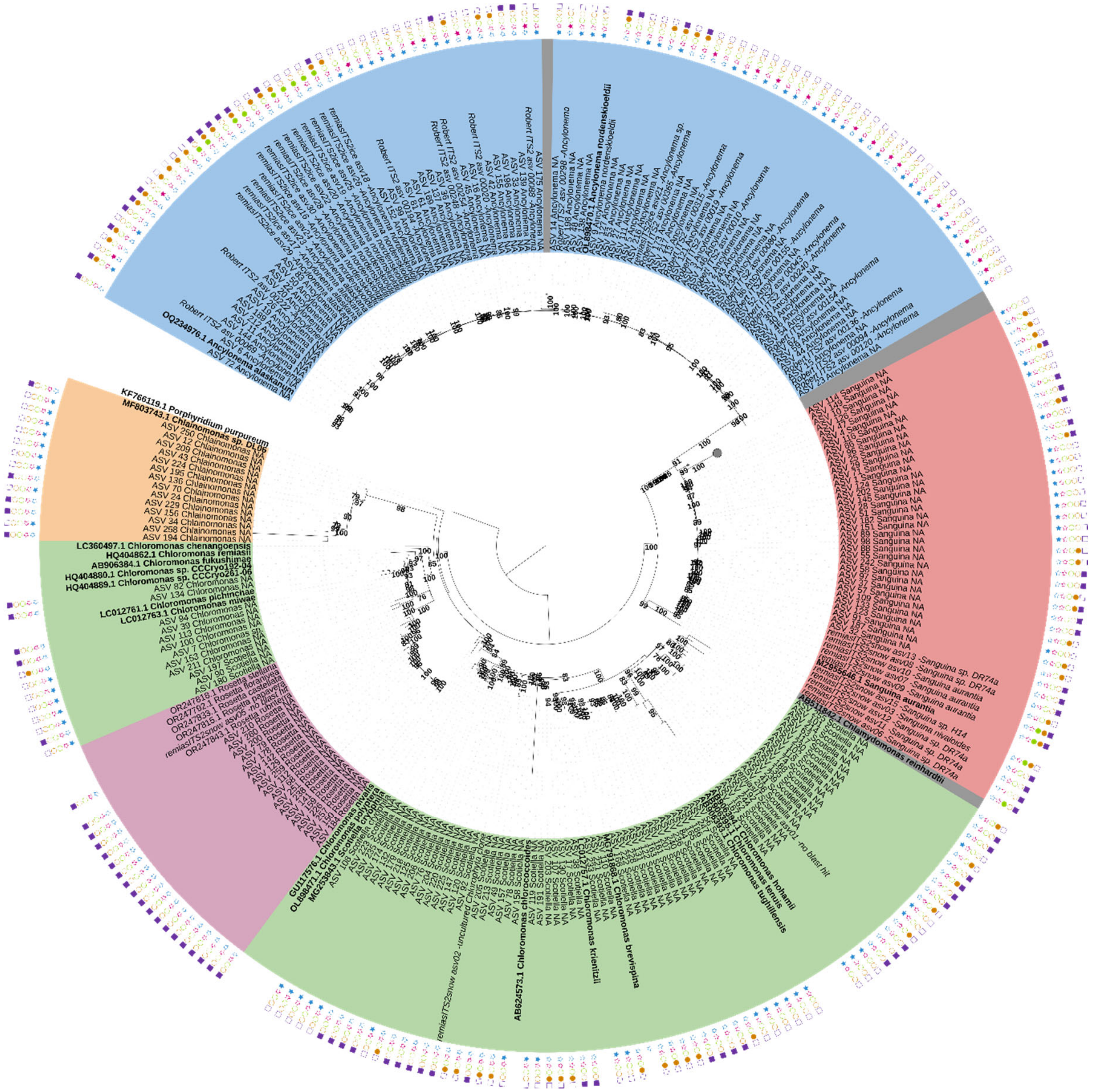

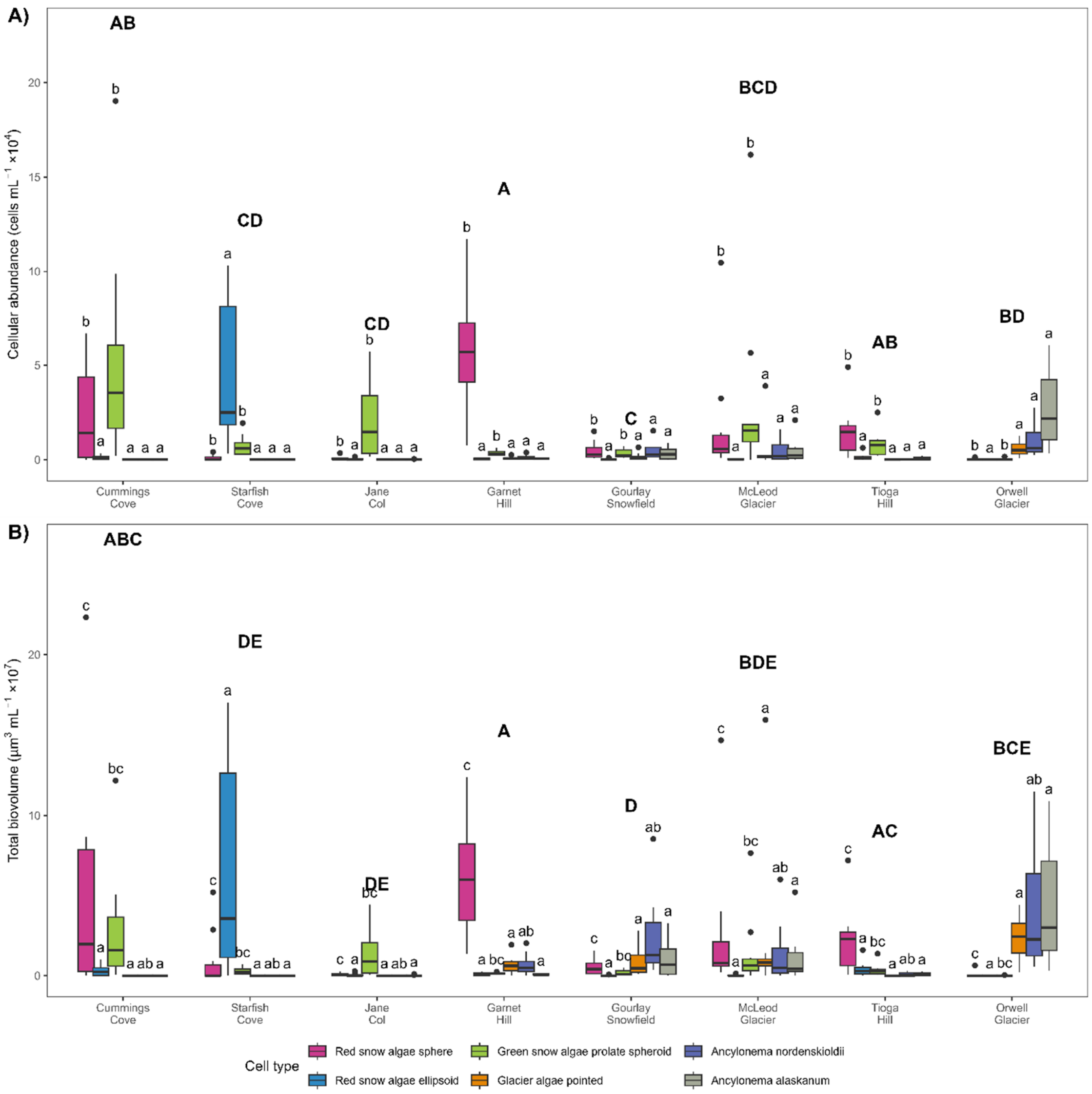


**Supplementary Figure 2:** Cellular abundance (cells mL$^{-1}$) and total biovolume (μm$^{3}$ mL$^{-1}$) of the snow and glacier algal species present as determined by average biovolume per cell per species and cellular abundance. Boxplots show median, interquartile range, and minimum to maximum values, with points showing potential outliers (N = 10). Lower case letters indicate homogenous subsets per species determined through a Kruskal Wallis test. **(A)** Cellular abundance (KW; $\chi^2 = 87.02$, $p < 0.01$), **(B)** Total biovolume (KW; $\chi^2 = 30.26$, $p < 0.01$). Upper case letters indicate homogenous subsets per site determined through a Kruskal Wallis test. **A)** Cellular abundance (KW; $\chi^2 = 38.49$, $p < 0.01$), **(B)** Total biovolume (KW; $\chi^2 = 52.75$, $p < 0.01$).

**Supplementary Figure 3:** Cellular abundance (cells $mL^{-1}$) and total biovolume ($\mu m^3$ $mL^{-1}$) of the snow and glacier algal species present as determined by average biovolume per cell per species and cellular abundance across 40 days on **A) Garnet Hill** (day 1 = 13/02/2024) and **B) Gourlay Snowfield** (day 1 = 10/02/2024). Plots show mean value ± SD error bars (n = 10).

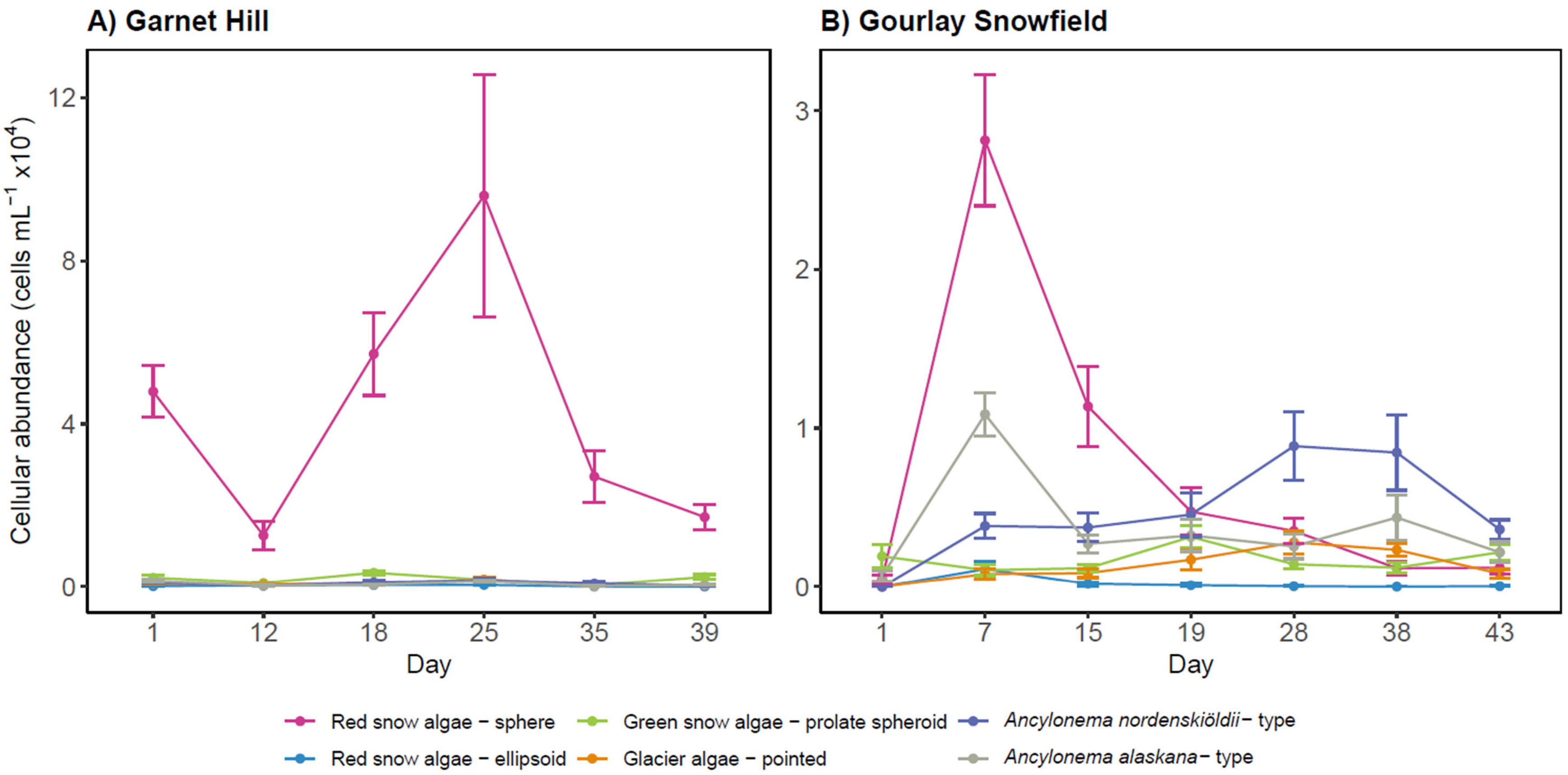

**Supplementary Figure 4:** Photos across the sampling season showing the progression of habitat conditions at **A) Garnet Hill** and **B) Gourlay Snowfield**.

**A) Garnet Hill**

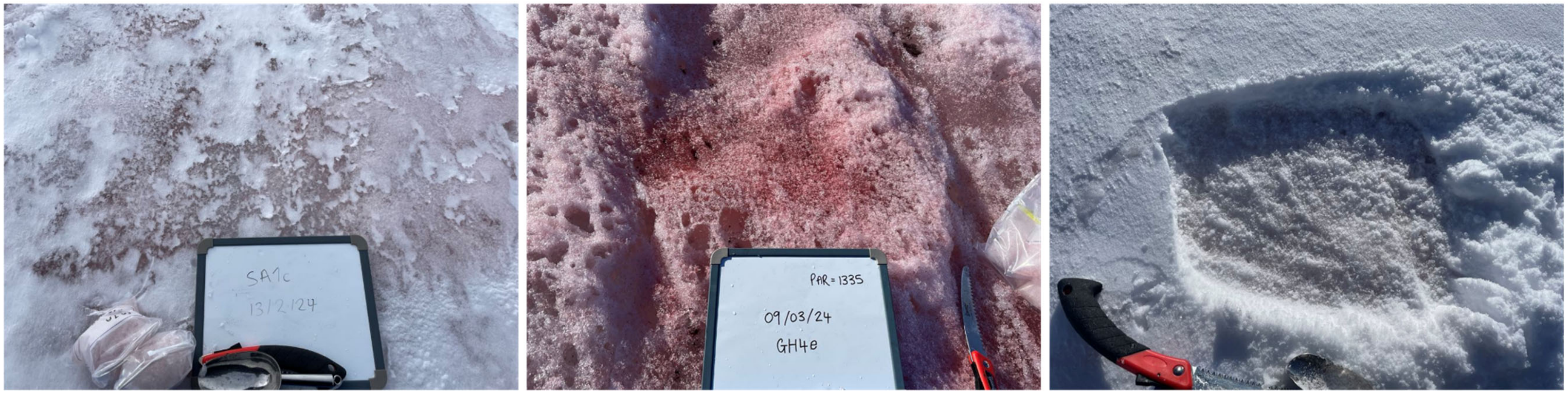


13/02/2024 – Day 1 | 09/03/2024 – Day 25 | 24/03/2024 – Day 39

**B) Gourlay Snowfield**

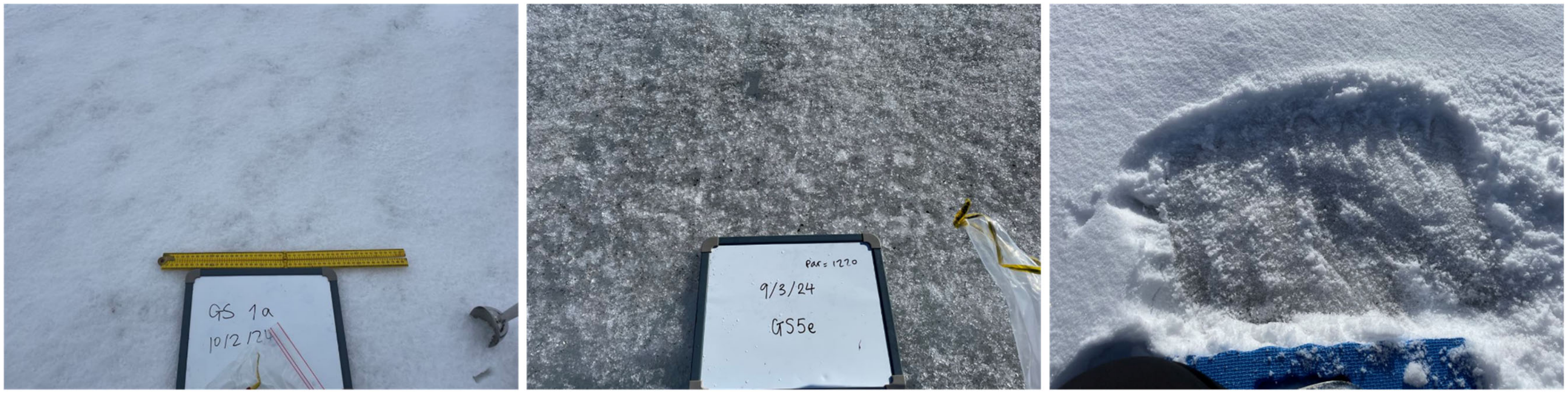


10/02/2024 – Day 1 | 09/03/2024 – Day 28 | 24/03/2024 – Day 43

**Supplementary Figure 5:** Bulk phase characterisation of inorganic and organic macro-nutrients over the start, middle, and end of the (A) Garnet Hill and (B) Gourlay Snowfield sampling period. Boxplots show median, interquartile range, and minimum to maximum values, with points showing potential outliers (N = 10). Lowercase letters indicate homogenous subsets determined through a Kruskal Wallis test. Lowercase letters indicate homogenous subsets determined through a Kruskal Wallis test. Garnet Hill: (A) Ammonium, (B) Nitrate (KW; $\chi2 = 59.69$, $p < 0.01$), (C) Nitrite (KW; $\chi2 = 8.48$, $p < 0.05$), (D) Phosphate, (E) DOC (F) DON (KW; $\chi2 = 7.96$, $p < 0.05$). Gourlay Snowfield: (A) Ammonium, (B) Nitrate, (C) Nitrite (KW; $\chi2 = 12.13$, $p < 0.01$) (D) phosphate, (E) DOC (KW; $\chi2 = 12.61$, $p < 0.01$), (F) DON.

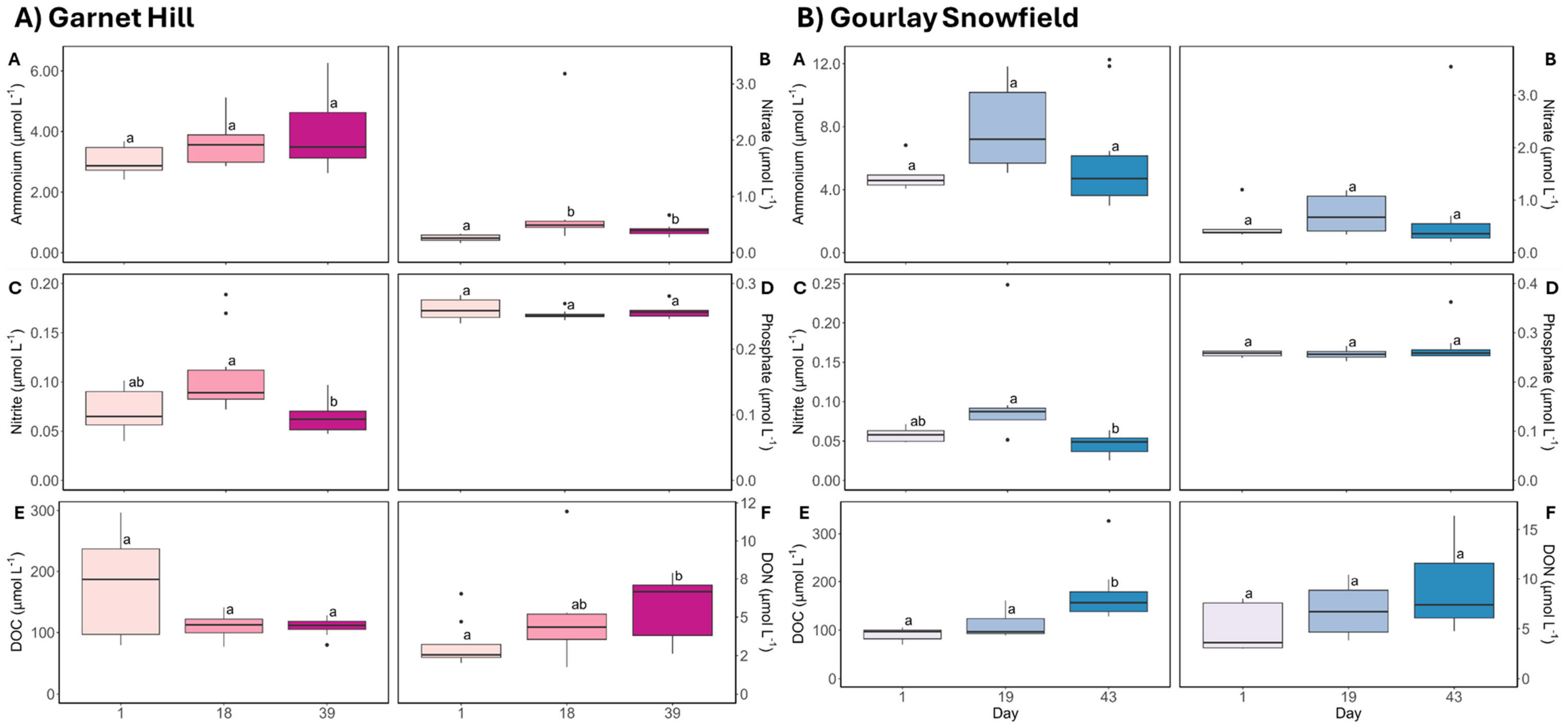

**Ambient geochemistry analysis**

*Data included in raw data upload.*

Aqueous concentrations of $NH_4^+$, $NO_3^+$, $NO_2^-$, and $PO_4^{3-}$ were derived for all melted ice and snow samples spectrophotometrically using a Gallery Plus Discrete Photometric Analyser (Thermo Fisher Scientific, UK). The limit of detection (LoD) for all nutrients was determined by the mean concentration plus three times the standard deviation of calibration blanks (N = 3). LoDs were 0.1 μmol $L^{-1}$ ($NH_4^+$), 0.02 μmol $L^{-1}$ ($NO_2^-$), 0.18 μmol $L^{-1}$ −($NO_3^-$), and 0.18 μmol $L^{-1}$ ($PO_4^{3-}$). Precisions were ±0.9% ($NH_4^+$), ±0.5% ($NO_2^-$), ±1.9% ($NO_3^-$), and ±1.6% ($PO_4^{3-}$) as determined by comparison with diluted 71.43 mmol $L^{-1}$ $NH_4^+$-N, $NO_2^-$-N, and $NO_3^-$-N and diluted 32.29 mmol $L^{-1}$ $PO_4^{3-}$-P certified stock standards to a concentration of 3.6 μmol $L^{-1}$ ($NH_4^+$ and $NO_2^-$), 2.9 μmol $L^{-1}$ ($NO_3^-$), and 6.1 μmol $L^{-1}$ ($PO_4^{3-}$) (Sigma TraceCERT®).

Filtrate was also analysed for total organic carbon (TOC) and total nitrogen (TN) concentrations via a TOC/TN Organic Carbon Analyser (Shimadzu, UK). Non-purgeable organic carbon (NPOC) was measured after the acidification of samples with 9N sulphuric acid and catalytic combustion at 720 °C as $CO_2$. TN was measured as NO by chemiluminescence. The LoD was 13.43 μmol $L^{-1}$ (TOC) and 34.5 μmol $L^{-1}$ (TN), precision was ±1.7% as determined by comparison with diluted 41.7 mmol $L^{-1}$ TOC certified stock standards to a concentration of 2.5 mmol $L^{-1}$ and ±1.2% by comparison with diluted 14.3 mmol $L^{-1}$ TN certified stock standards to a concentration of 3.6 mmol $L^{-1}$ (Sigma TraceCERT®).

**References**


Cheung, M.K. *et al.* (2010) 'Composition and genetic diversity of picoeukaryotes in subtropical coastal waters as revealed by 454 pyrosequencing', *The ISME Journal*, 4(8), pp. 1053–1059. Available at: https://doi.org/10.1038/ismej.2010.26.

Innis, M.A. *et al.* (1990) *PCR Protocols: A Guide to Methods and Applications*. Academic Press.

McCutcheon, J. *et al.* (2021) 'Mineral phosphorus drives glacier algal blooms on the Greenland Ice Sheet', *Nature Communications*, 12(1), p. 570. Available at: https://doi.org/10.1038/s41467-020-20627-w.

Mikhailyuk, T.I. *et al.* (2008) 'New Streptophyte Green Algae from Terrestrial Habitats and an Assessment of the Genus Interfilum (klebsormidiophyceae, Streptophyta)', *Journal of Phycology*, 44(6), pp. 1586–1603. Available at: https://doi.org/10.1111/j.1529-8817.2008.00606.x.

Remias, D. *et al.* (2023) 'Novel insights in cryptic diversity of snow and glacier ice algae communities combining 18S rRNA gene and ITS2 amplicon sequencing', *FEMS Microbiology Ecology*, 99(12), p. fiad134. Available at: https://doi.org/10.1093/femsec/fiad134.

Smith, H.J. *et al.* (2017) 'Microbial formation of labile organic carbon in Antarctic glacial environments', *Nature Geoscience*, 10(5), pp. 356–359. Available at: https://doi.org/10.1038/ngeo2925.

Thomson, A.I. *et al.* (2025) 'Surface darkening by abundant and diverse algae on an Antarctic ice cap', *Nature Communications*, 16(1), p. 2647. Available at: https://doi.org/10.1038/s41467-025-57725-6.

Wadham, J.L. *et al.* (2016) 'Sources, cycling and export of nitrogen on the Greenland Ice Sheet', *Biogeosciences*, 13(22), pp. 6339–6352. Available at: https://doi.org/10.5194/bg-13-6339-2016.
